# miR‐505 inhibits proliferation of osteosarcoma via HMGB1

**DOI:** 10.1002/2211-5463.12868

**Published:** 2020-05-31

**Authors:** Guangzhang Li, Fajing Liu, Jun Miao, Yongcheng Hu

**Affiliations:** ^1^ The Graduate School Tianjin Medical University China; ^2^ Department of Spine Surgery Tianjin Hospital China; ^3^ Department of Orthopedic Oncology Tianjin Hospital China

**Keywords:** apoptosis, biological functions, HMGB1, miR‐505, osteosarcoma

## Abstract

Osteosarcoma is a malignant bone tumor, and clinically detectable metastases can be detected in ~ 15–20% of patients when they seek medical advice; patients with metastatic disease have extremely poor prognosis. Here, we examined the involvement of the microRNA miR‐505 in osteosarcoma. Eighty‐four patients seeking treatment for osteosarcoma were included in the study group (SG), and 63 healthy subjects were allocated to the control group (CG). Normal human bone cells MG‐63 and U20S cells were transfected with miR‐505 mimics, miR‐NC, HMGB1 RNA for targeted inhibition (si‐HMGB1), and si‐NC to examine the effects on HMGB1 expression. Cell proliferation, invasion, and apoptosis were measured using CCK‐8, scratch assays, and flow cytometry (FCM), respectively, and the relationship between miR‐505 and HMGB1 was determined using the dual‐luciferase reporter assay. In patient tissues and serum, miR‐505 was expressed at a low level, and HMGB1 was expressed at a high level, with an area under curve of > 0.9. Furthermore, the expression of miR‐505 and HMGB1 in tissues had a positive association with that in the serum, whereas the expression of miR‐505 had a negative association with that of HMGB1 in tissues only. Overexpression of miR‐505 and silencing of HMGB1 suppressed the proliferation, migration, and invasion of osteosarcoma cells and increased the rate of apoptosis, whereas the co‐transfected miR‐505 mimics + si‐HMGB1 demonstrated a more significant inhibitory effect on the proliferation and invasion of osteosarcoma cells and a higher apoptosis rate. miR‐505 may inhibit the proliferation and invasion and promote apoptosis of osteosarcoma cells by targeting and suppressing HMGB1.

## Graphical abstract

miRs are associated with tumor progression, and miR‐505 may act as a tumor suppressor in certain tumor types. Low expression of mir‐505 and high expression of HMGB1 were observed in osteosarcoma tumor cells. It was found that overexpression of mir‐505 inhibited the expression of HMGB1 and restricted the growth of osteosarcoma tumor cells.

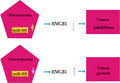

**Abbreviations**
AUC: area under curve
FCM: flow cytometry
miR: microRNA
miR‐NC: miR negative control
si‐HMGB1: HMGB1 RNA for targeted inhibition
WB: western blot

The primary osteocarcinoma, osteosarcoma [1], is a malignant bone tumor [2, 3, 4] affecting children, teenagers, and young men. It originates from mesenchymal cells, preferentially develops at the metaphysis of the long bone, and results in sharp pain or other vital signs related to pathologic fracture. According to some studies, clinically detectable metastases can be found in ~ 15–20% of patients when they seek medical advice [5]; patients with metastatic disease may have an extremely poor prognosis or those with a possibility of reoccurrence have an extremely poor prognosis. For this, new views are necessary [6] to explain the mechanisms related to osteosarcoma and find potential targets to improve the prognosis of patients with osteosarcoma.

microRNA (miR) accounts for tumor progression in majority of cancer types [7]. In recent years, the abnormal expression of miR in tumors has been observed in a number of studies; consequently, miR has been suggested as a tumor target [8, 9, 10] given its role in supporting the functions of cancer genes and anti‐oncogenes. Being a member of miR, the possible role of miR‐505 as an anti‐oncogene has been revealed in several types of tumors in recent years [11, 12]. Some studies have shown that miR‐505 can promote the chemoresistance of mammary cancer cells by inhibiting cell proliferation through apoptosis induction and its expression is related to established biomarkers for the prognosis of mammary cancer [13].

HMGB1 is a highly abundant protein with extensive expression; it is actively secreted in immunological cells or passively released in injured or dying cells, thus acting as an extracellular signal [14, 15, 16]. Furthermore, extracellular HMGB1 can trigger and maintain inflammatory reactions by inducing the release of cellular factors and recruiting white blood cells; thus, it has been used as a key molecular target in many diseases [17]. According to the study by Martinotti *et al*. [18], HMGB1 enhanced the proliferation, migration, and osteogenic differentiation of SaOS‐2 cells, indicating that it may represent an effective bone remodeling signal. In the study by Tian *et al*. [19], miR‐505 was shown to suppress lung cancer progression by targeting HMGB1, highlighting that both miR‐505 and HMGB1 participate in lung cancer development.

To date, there are limited studies focusing on the mechanisms of miR‐505 and HMGB1 with respect to osteosarcoma. Therefore, in this study, we have discussed the expression of miR‐505 and HMGB1in the tissues of patients with osteosarcoma and its effect on the biological functions of osteosarcoma cells.

## Materials and methods

### General materials

In total, 84 patients admitted to the Department of Surgical Oncology from January 2017 to February 2019 owing to osteosarcoma were included in the SG; the SG included 45 males and 39 females, of whom 52 were aged < 20 years and 32 were ≥ 20 years. In addition, 63 healthy subjects who underwent physical examination in our hospital during the same period were included to the control group (CG); the CG included 33 males and 30 females, of whom 35 were aged < 20 years and 28 were ≥ 20 years. All clinical experimental indexes and imaging results of the CG were normal. The guardians of patients in this study provided written informed consent after understanding the study, which complied with local ethics and morality. The proposal was reviewed and approved by the Ethics Committee of Tianjin Hospital prior to implementation.

### Inclusion and exclusion criteria

The inclusion criteria were as follows: patients in the SG who were diagnosed with osteosarcoma via pathological examination and X‐ray images [20], with an expected survival over 3 months; they had not received any radiotherapy or chemotherapy. The exclusion criteria were as follows: presence of concurrent malignant tumors, metal disorders, or conditions, which rendered them unsuitable for the study at the discretion of the researchers. Patients who failed to cooperate with the surgery and treatment or those who were pregnant or lactating were also excluded. The study methodologies conformed to the standards set by the Declaration of Helsinki.

### Sources of cells

Normal human bone cells hFOB1.19 (Shanghai Huzhen Biotechnology Co., Ltd., product no.: HZ‐H321), osteosarcoma 143B cells (Shenzhen Otwo Biotech Co., Ltd., Shanghai, China, product no.: HTX‐2774), osteosarcoma MG‐63 cells (Shenzhen Otwo Biotech Co., Ltd., Shenzhen, China, product no.: HTX‐1630), osteosarcoma U20S cells (Shanghai Kaming Biotechnology Co., Ltd., Shanghai, China, product no.: Sci‐M215), and osteosarcoma HOS cell (Shanghai Xuanya Biotechnology Co., Ltd., Shanghai, China, product no.: XY‐XB‐1716) were used in the current study.

### Sources of reagents and instruments

CCK‐8 (Bestbio, Shanghai, China, BB‐4202); Dulbecco’s modified Eagle’s medium (DMEM; Shanghai Yuanye Biotechnology Co., Ltd., Shanghai, China, R20163); PBS, BFS, and penicillin–streptomycin (Gibco, Big Island, NY, USA, 10010023, 26400044, and 15070063, respectively); RIPA reagent, bicinchoninic acid protein kit, and ECL chemiluminescence kit (Shanghai Guyan Industry Co., Ltd., Shanghai, China, GOY‐0839P, GOY‐0675SJ, and GOY‐0532P, respectively); parenzyme (Shanghai Huiying Biotechnology Co., Ltd., Shanghai, China, 25200‐056); Lipofectamine™ 2000 Transfection Reagent (Shanghai Mituo Biotechnology Co., Ltd., Shanghai, China, 11668019); SYBR Green PCR Master Mix (Applied Biosystems, Waltham, MA, USA); sex‐determining region Y box (SOX1), β‐actin first antibody, and HRP‐labeled goat anti‐mouse IgG secondary antibody (R&D, Minneapolis, MN, USA, AF4196, MAB8929, and HAF007, respectively; HMGB1 (Shanghai Hengfei Biotechnology Co., Ltd., K003412P); TRIzol extraction kit (Wuhan Chundu Biotechnology Co., Ltd., China, CDLG‐4396); reverse transcription kit [Tiangen Biotech (Beijing) Co., Ltd., Beijing, China, FP209]; Annexin V/PI apoptosis kit (Shanghai Yusheng Biotechnology Co., Ltd., Shanghai, China, 40302ES20); and dual‐luciferase reporter gene assay kit (Beijing Solarbio Co., Ltd., Beijing, China, D0010) were used in the current study.

A PCR instrument (Beijing Pengkun Boyuan Technology Trading and Development Co., Ltd., Beijing, China, T15899), a flow cytometer (Thermo Fisher Scientific (Shanghai) Co., Ltd., China (Shanghai) Pilot Free Trade Zone, A25750), and an ELISA reader (Shanghai Bajiu Industry Co., Ltd., Shanghai, China SAF‐680T) were used throughout the course of the research. Moreover, all the primers were designed and synthesized by Suzhou Jinweizhi Biotechnology Co., Ltd (Suzhou, China; Table 1).

**Table 1:** List of primer sequence.

Gene name	Sequence
hsa‐miR‐505 mimic	Forward 5 ′ ‐GGGAGCCAGGAAGUAUU GAUGU‐3 ′
	Reverse 5′‐ACAUCAAUACUUCCUGGCUCUU‐3′
hsa‐miR‐505 inhibitor	5 ′ ‐GGGAGCCAGGAAGU AUUGAUGU‐3 ′
miRNA mimic control sequence	Forward 5′‐UUCUCCGAACGUGUCACGU TT‐3′
	Reverse 5′‐ACGUGACACGUUCGGAGAATT‐3′
miRNA inhibitor control sequence	5′‐CAGUACUUUU GUGUAGUACAA‐3′
HMGB1‐qPCR	Forward 5′‐TTCAAGGAAGAGAAAACAACAA‐3′
	Reverse 5′‐ATGGCAGGTATTATTAAGGAGG‐3′
U6 RT primer	5′‐CGCTTCACGAATTTGCGTGTCAT‐3′
U6‐PCR primer	Forward 5′‐GCTTCGGCAGCACATATACTAAAAT‐3′
	Reverse 5′‐CGCTTCACGAATTTGCGTGTCAT‐3′
GAPDH‐PCR primer	Forward 5'‐GGAGC‐GAGATCCCTCCAAAT‐3AT
	Reverse GGCTGTTGTCATACTTCTCATGG‐3'

### Sample collection

Following provision of written informed consent from patients and the hospital, cancerous and paracancerous tissues were harvested from 84 patients with osteosarcoma and stored in liquid nitrogen. In addition, 5 mL of blood was drawn from the ulnar veins of the patients in the SG and CG and centrifuged for 10 min at 3000 ***g***. The serum was reserved for future study.

### Cell cultivation and transfection

Purchased cells were transferred to low‐sugar DMEM containing penicillin, streptomycin, and 10% FBS and cultivated in an incubator at 37 °C with 5% CO_2_. With groups including miR‐505 mimics (overexpression sequence), miR negative control (miR‐NC), HMGB1 RNA for targeted inhibition (si‐HMGB1), and RNA for a negative control, cells were transinfected using a Lipofectamine™ 2000 kit according to the manufacturer’s instructions. The cells with the highest difference in the expression were transinfected by primers.

### qRT‐PCR detection

Total RNA extraction of cells, tissues, and serums was conducted using a TRIzol kit, and the purity, concentration, and integration were tested using an ultraviolet spectrophotometer and agarose gel electrophoresis. Reverse transcription of miR‐505 and HMGB1 was performed in strict accordance with the instructions provided with the kits, followed using a PCR reaction system of 2 × Talent qPCR PreMix 10 µL, with 1.25 µL upstream and downstream primers, respectively, and 100 ng cDNA. The PCR reaction conditions included a predenaturation step at 94 °C for 30 min, denaturation at 95 °C for 5 s, annealing and extension at 60 °C for 30 s, with a total of 40 cycles. U6 and GAPDH were adopted as the internal reference controls of miR and mRNA, respectively. Data were analyzed by $2^{-\Delta \Delta C_{t}}$.

### Western blot (WB)

The total protein was extracted from the cultivated cells of each group using RIPA lysis buffer, and the protein concentration was measured by bicinchoninic acid. The protein concentration was adjusted to 4 μg·μL^−1^, and iontophoretic separation was performed with 12% SDS/PAGE, after which the membrane was transferred to PVDF, stained with Ponceau S, soaked and rinsed in PBST for 5 min, and sealed with 5% dried skimmed milk for 2 h. The primary antibodies (SOX1 and β‐actin) were then added at a dilution of 1 : 1000 and sealed standing overnight at 4 °C. The next day, the membrane was rinsed to remove the primary antibody, and the horseradish peroxidase‐labeled goat anti‐rabbit secondary antibody was added at a dilution of 1 : 5000 for 1 h incubation at 37 °C; this was followed by three washes with PBS, each lasting for 5 min. Development was performed in a dark room, and excessive liquid on the membrane was absorbed with filter paper, and ECL was used to develop the image. The protein strip was scanned to analyze the gray value using the quantity one software (Bio‐rad, Irvine, CA, USA), and the relative expression of protein was equal to the gray value of the target protein strip/gray value of the β‐actin protein strip.

### Cell proliferation detection with CCK‐8

Cells transfected for 24 h were collected, adjusted to 4 × 10^6^, and then incubated in a 96‐well plate for 24, 48, 72, and 96 h; 10 μL CCK solution was added to each well; and 90 μL was added to the DMEM for cultivation for 2 h at 37 °C. The absorbance of each group was measured by an ELISA reader at a wavelength of 490 nm.

### Detection of cell invasion (transwell)

Following transfection for 24 h, cells were collected, adjusted to 5 × 10^4^, and plated in a 6‐well plate before rinsing twice with PBS and incubating the upper chamber. Then, 200 μL of DMEM was added to the upper chamber, whereas 500 mL of DMEM (containing 20% FBS) was added to the lower chamber, followed by culturing at 37 °C for 48 h. The substrate and cells that did not penetrate through the membrane surface were then wiped, rinsed with PBS, and fixed with paraformaldehyde for 10 min. Subsequently, they were rinsed three times with double‐distilled water and dried, followed by dyeing with 0.5% crystal violet; finally, cell invasion was observed under the microscope.

### Detection of cell apoptosis by flow cytometry (FCM)

Transfected cells were digested by 0.25% parenzyme and then rinsed twice with PBS before adding 100 μL of binding buffer to harvest the suspension with 1 × 10^6^ cells per mL, into which Annexin V‐FITC and PI were added in sequence. The solution was then incubated for 5 min at 37 °C in the dark. Cells were analyzed by FCM, and the mean of values of the three experiments performed in triplicates was calculated.

### Detection of luciferase activity

The downstream target gene of miR‐505 was predicted by Targetscan7.2. HMGB1 3′ UTR‐Wt, HMGB1 3′ UTR‐Mut, miR‐505a mimics, and miR‐NC were transferred to MG‐63 and U20S cells using a Lipofectamine™ 2000 kit. The dual‐luciferase reporter assay was performed to detect the luciferase activity.

### Statistical analysis

Statistical analysis was performed, and the related drawings were drawn using the spss 22.0 software (SPSS, Stanford, CA, USA). In case of nominal data, expressed as [*n* (%)], intergroup comparison studies were conducted via the χ^2^ test. In case of numerical data, expressed as mean ± standard deviation, intergroup comparison studies were conducted via independent samples *t*‐test. One‐way analysis of variance (ANOVA) was used for comparison among groups. ROC was used to evaluate the diagnostic values of miR‐505 and HMGB1 in osteosarcoma, and Pearson’s test was used to determine the correlation of tissues and serum in terms of miR‐505 and HGB1. For all statistical comparisons, a *P* value of < 0.05 was considered to be statistically significant.

## Results

### Expression of miR‐505 and HMGB1 in osteosarcoma tissues and serum

**Fig. 1:**
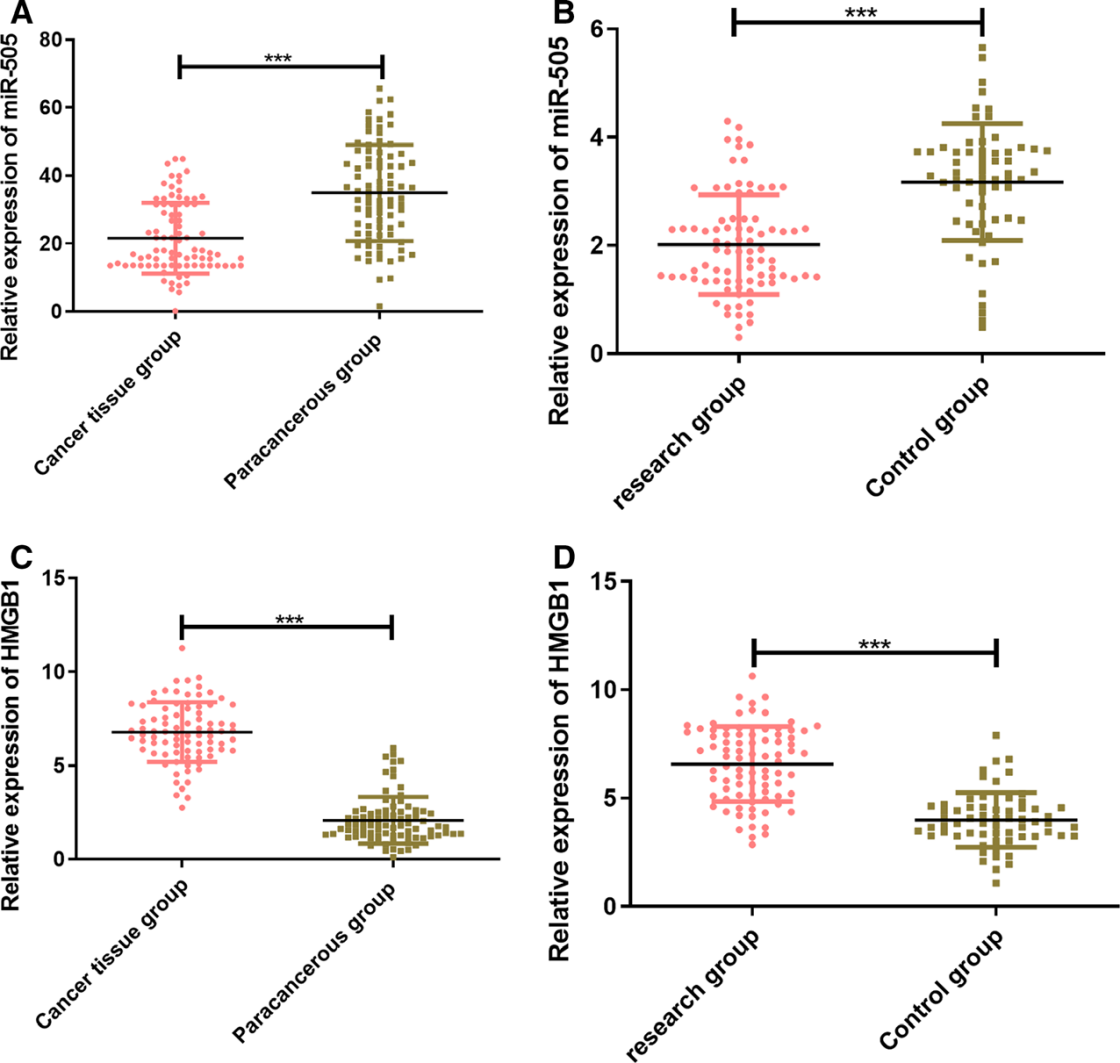
Expression of miR‐505 and HMGB1 in osteosarcoma tissues and serum. The data of this study in this figure are all expressed in terms of measurement data and represented by mean ± standard deviation (mean ± SD). The test methods were independent sample t‐test. In the cancer tissues of patients with osteosarcoma, the expression of miR‐505 was significantly reduced as compared with that of paracancerous tissues (A). In the cancer serum of patients with osteosarcoma, the expression of miR‐505 was significantly reduced as compared with that in the CG (B). In the cancer tissues of patients with osteosarcoma, the expression of HMGB1 was significantly increased as compared with that of paracancerous tissues (C). In the cancer serum of patients with osteosarcoma, the expression of HMGB1 was significantly increased as compared with that in the CG (D). Compared with the CG, ^***^ *P* < 0.001.

According to the qRT‐PCR results, the relative expression of miR‐505 and HMGB1 in the tissues and serum of the patients in the SG were lower and higher than the CG, respectively (*P* < 0.001; Fig. 1).

### Clinical diagnostic values of miR‐505 and HMGB1 in osteosarcoma cells

**Fig. 2:**
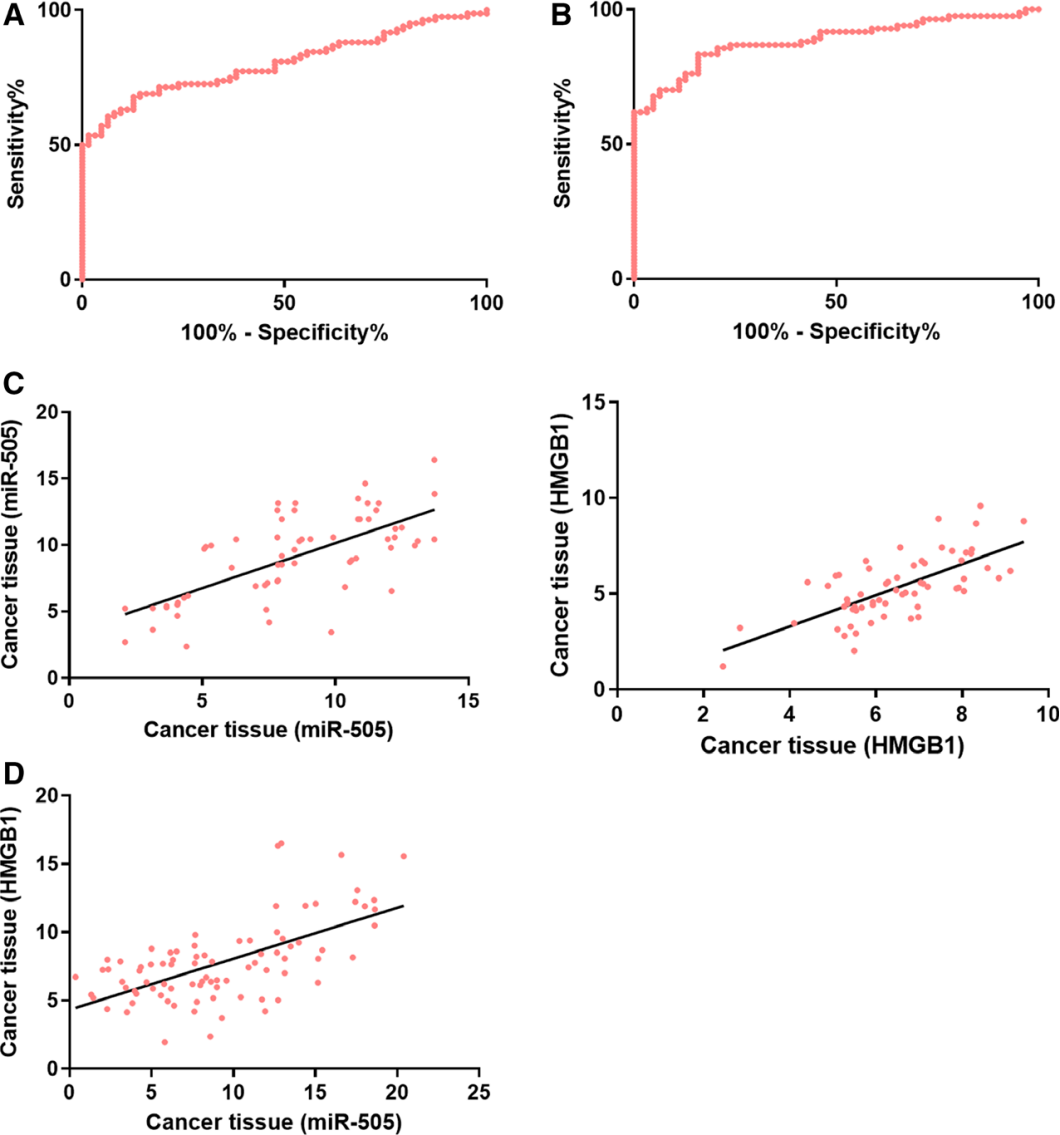
Clinical diagnostic values of miR‐505 and HMGB1 in osteosarcoma cells. The sensitivity and specificity of osteosarcoma diagnosis by serum miR‐505 and HMGB1 measurements were 67.86% and 87.30% (A) and 83.33% and 84.13% (B), respectively. A positive relationship was established between the expression of miR‐505 and HMGB1 in cancer tissues and serum (*r* = 0.677, *P* < 0.001; *r* = 0.692, *P* < 0.001, respectively) (C), and a negative relationship was observed between the expression of miR‐505 and HMGB1 in cancer tissues (*r* = 0.616, *P* < 0.001) (D).

The best cutoff value, sensitivity, and specificity were determined according to the ROC of osteosarcoma diagnosis based on serum miR‐505 and HMGB1 expression. In the case of miR‐505, these values were 9.79 pg·mL^−1^, 67.86%, and 87.30%, respectively, with an area under curve (AUC) of 0804; in the case of HMGB1, these values were 5.09 ng·mL^−1^, 83.33%, and 84.13%, respectively, with an AUC of 0.885. According to Pearson’s test, in tissues, the expression of miR‐505 was negatively associated with that of HMGB1 (*P* < 0.001). Further analysis of the pathological data and serum miR‐505 and HMGB1 expression found a close relationship among miR‐505, HMGB1, TNM staging, tumor size, and distant metastasis (Table 2 and Fig. 2).

**Table 2:** Relationships among miR‐505, HMGB1, and clinical pathological characteristics of patients with osteosarcoma.

Type	*n*	miR‐505		*t*	*P*	HMGB1		*t*	*P*
		High expression	Low expression						
Sex									
Male	45	27 (60.00)	18 (40.00)	2.256	0.133	29 (64.44)	16 (35.56)	2.110	0.146
Female	39	22 (56.41)	17 (43.59)			19 (48.72)	20 (51.28)		
Age (y)									
< 20	52	23 (44.23)	29 (55.771)	0.369	0.543	31 (59.62)	21 (40.38)	1.297	0.255
≥ 20	32	12 (37.50)	20 (62.50)			23 (71.88)	9 (28.13)		
Anatomical position									
Tibia/thighbone	60	18 (30.00)	11 (45.83)	1.901	0.168	44 (73.33)	16 (26.67)	0.311	0.577
Other parts	24	42 (70.00)	13 (54.17)			19 (79.17)	5 (20.83)		
TNM staging									
I + II stages	50	32 (64.00)	18 (36.00)	6.686	0.009	19 (38.00)	31 (62.00)	5.777	0.016
III + IV stages	34	12 (35.29)	22 (64.71)			22 (64.71)	12 (35.29)		
Tumor size (cm)									
≤ 7	35	21 (60.00)	14 (40.00)	10.791	0.001	15 (42.86)	20 (57.14)	4.175	0.041
> 7	49	12 (24.49)	37 (75.51)			32 (65.31)	17 (34.69)		
Distant metastasis									
Yes	29	11 (37.93)	18 (62.07)	7.585	0.006	18 (62.07)	11 (37.93)	9.639	0.002
No	55	38 (69.09)	17 (30.91)			15 (27.27)	40 (72.73)		
Histological type									
Routine type	69	34 (49.28)	35 (50.72)	0.034	0.855	45 (65.22)	24 (34.78)	0.146	0.702
Special type	15	7 (46.67)	8 (53.33)			9 (60.00)	6 (40.00)		

### Expression of miR‐505 in cells and its influence on the biological functions of osteosarcoma cells

**Fig. 3:**
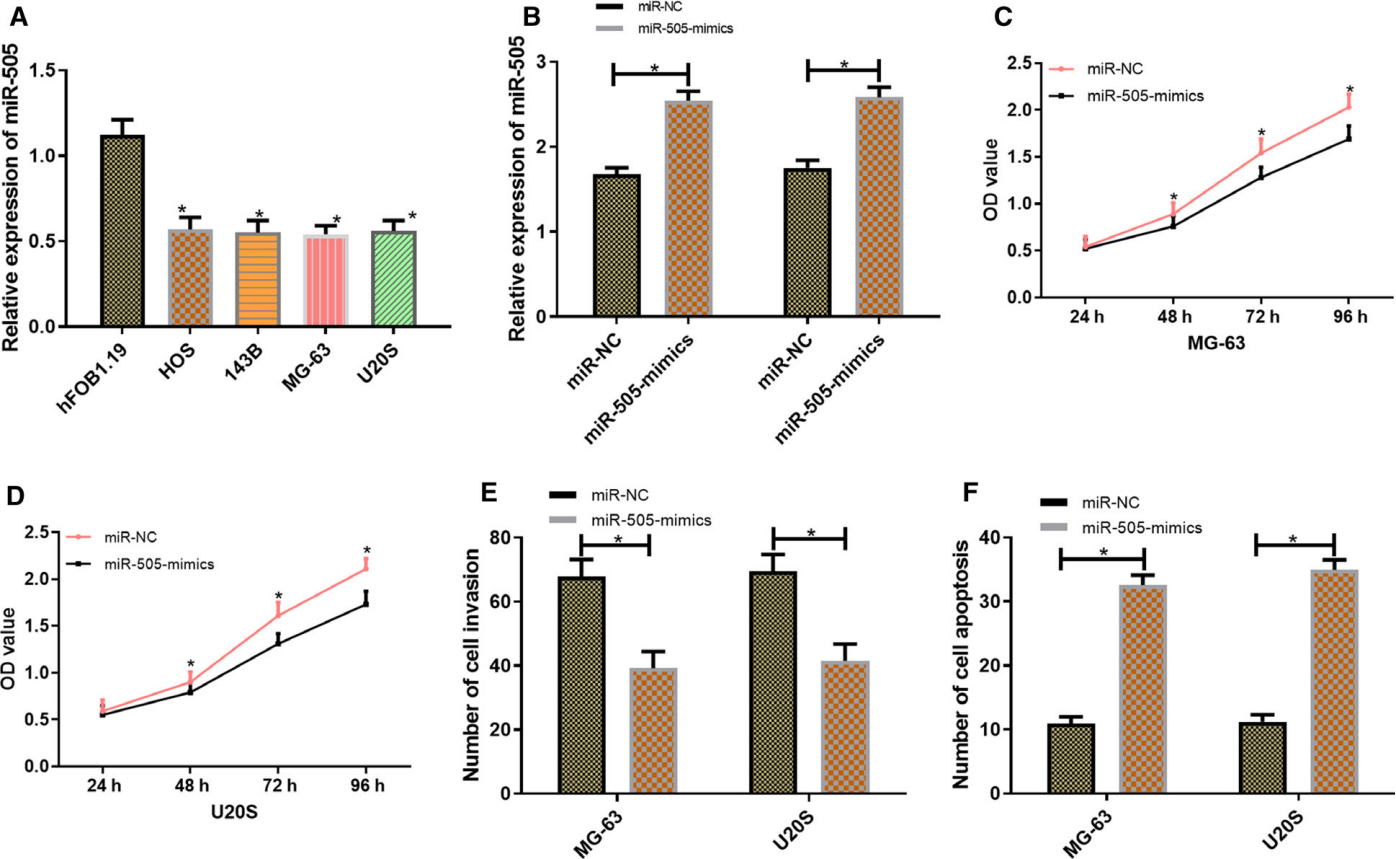
Expression of miR‐505 in cells and its influence on the biological functions of osteosarcoma cells. The data of this study in this figure are all expressed in terms of measurement data and represented by mean ± standard deviation (mean ± SD). The test methods were independent sample *t*‐test and one‐way ANOVA. *n* = 3. Expression of miR‐505 in the cells of each group (A). Expression of miR‐505 in transfected MG‐63 and U20S cells (B). Proliferation of transfected MG‐63 and U20S cells (C, D). Invasion capacity of transfected MG‐63 and U20S cells (E). Apoptosis of transfected MG‐63 and U20S cells (F). Compared with miR‐NC, *P < 0.05.

Detection of miR‐505 in the cells of each group demonstrated that miR‐505 expression was significantly lower in HOS, 143B, MG‐63, and U20S than hFOB1.19 cells in patients with osteosarcoma (*P* < 0.001). The MG‐63 and U20S cells with the greatest difference were selected for transfection to detect the expression of transfected miR‐505 mimics in cells. Results showed that the expression of miR‐505 was significantly higher in transfected miR‐505 mimics cells than that in miR‐NC cells (*P* < 0.001). Compared with miR‐NC, MG‐63 and U20S cells, after transfection with miR‐505 mimics, were inhibited in terms of proliferation and invasion, whereas their apoptosis rate rose sharply (*P* < 0.001; Fig. 3).

### Expression of HMGB1 in cells and its influence on the biological functions of cells in osteosarcoma

**Fig. 4:**
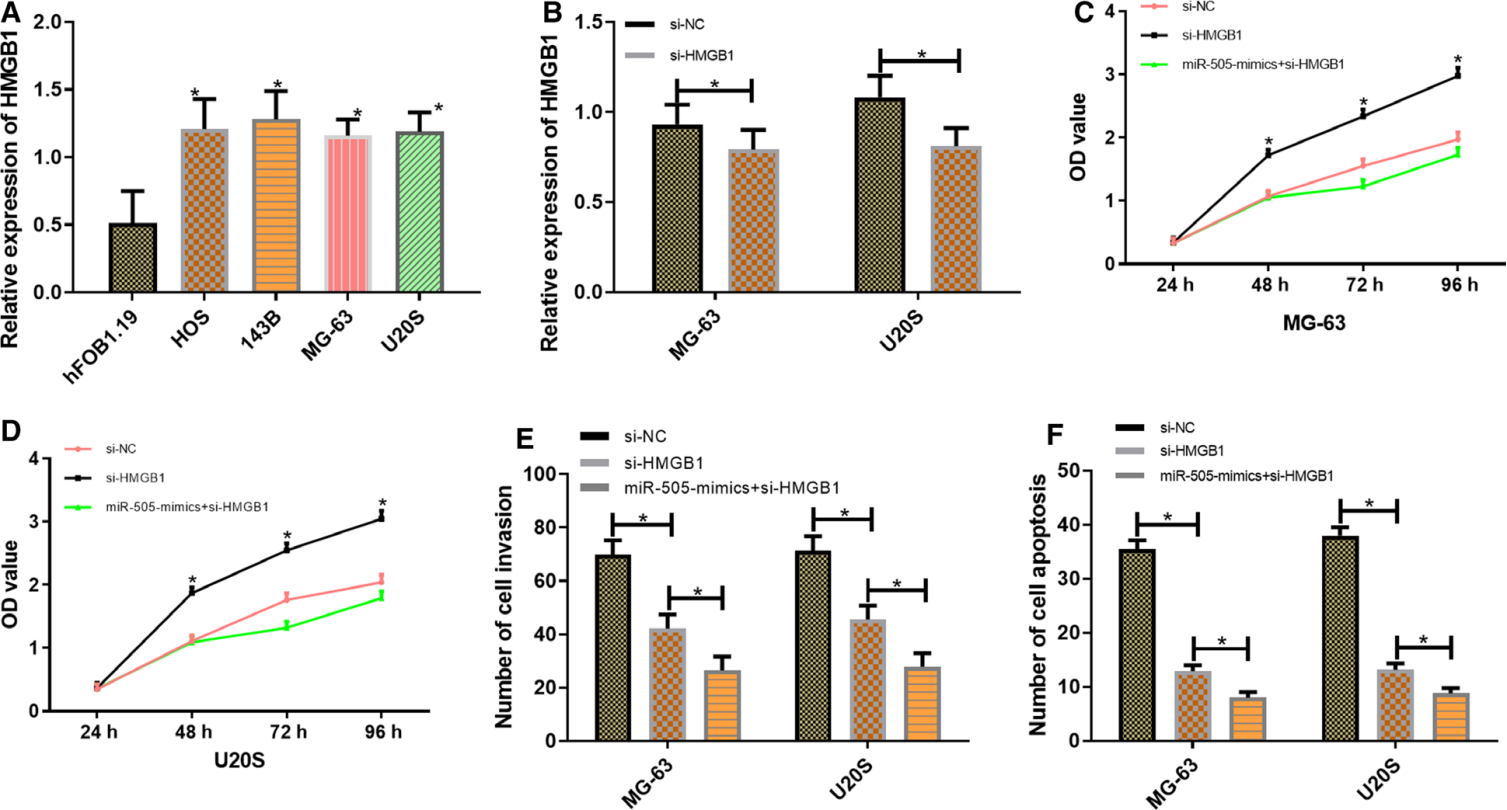
Expression of HMGB1 in cells and its influence on the biological functions of osteosarcoma cells. The data of this study in this figure are all expressed in terms of measurement data and represented by mean ± standard deviation (mean ± SD). The test methods were independent sample *t*‐test and one‐way ANOVA. *n* = 3. Expression of HMGB1 in the cells of each group (A). Expression of HMGB1 in transfected MG‐63 and U20S cells (B). Proliferation of transfected MG‐63 and U20S cells (C, D). Invasion capacity of transfected MG‐63 and U20S cells (E). Apoptosis of transfected MG‐63 and U20S cells (F). Compared with the miR‐NC, *P < 0.05.

The expression of HMGB1 in HOS, 143B, MG‐63, and U20S cells was significantly higher than that in hFOB1.19 cells in patients with osteosarcoma (*P* < 0.001). MG‐63 and U20S cells with the greatest difference were selected for transfection to detect the expression of transfected si‐HMGB1. Results showed that the expression of HMGB1 in transfected si‐HMGB1 cells was lower than that in si‐NC cells (*P* < 0.001). Compared with si‐NC, MG‐63 and U20S cells, after transfection with si‐HMGB1, demonstrated lower proliferation and invasion capacity and a higher apoptosis rate (*P* < 0.001; Fig. 4).

### miR‐505 gene identification

**Fig. 5:**
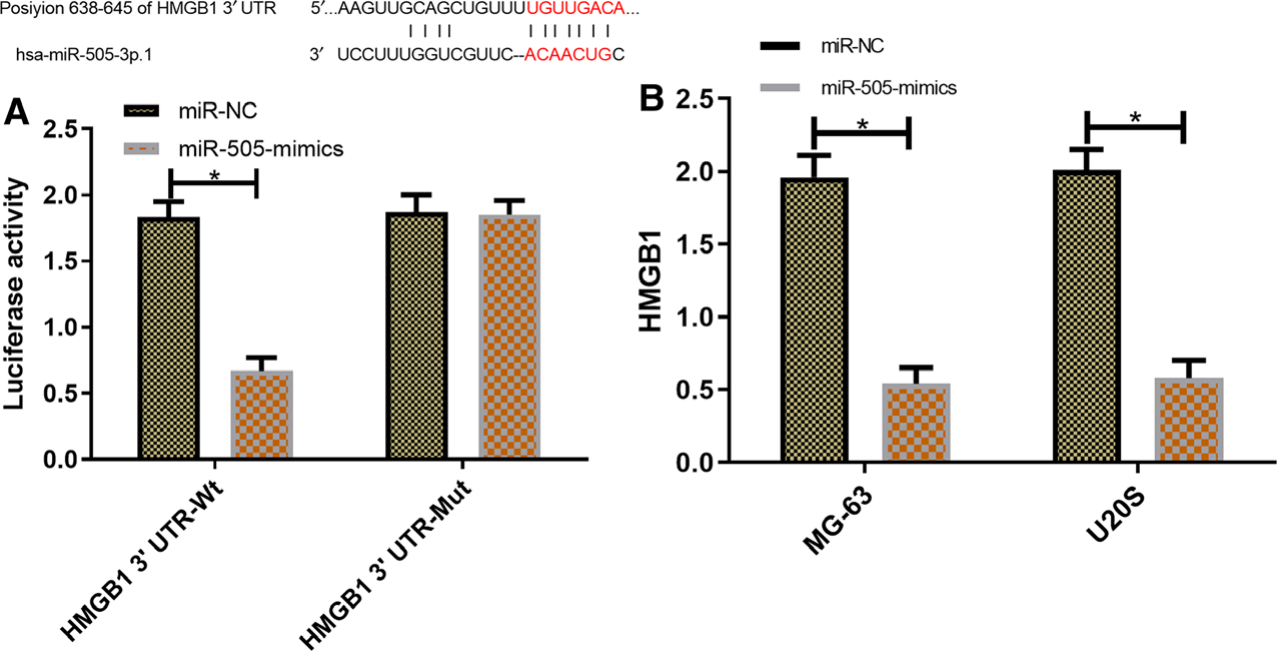
miR‐505 gene identification. The data of this study in this figure are all expressed in terms of measurement data and represented by mean ± standard deviation (mean ± SD). The test methods were independent sample *t*‐test. *n* = 3. Binding loci between miR‐505 and HMGB1 and results of relative luciferase activity and dual‐luciferase activity (A). Relative expression of HMGB1 in MG‐63 and U20S cells after transfection (B). Compared with the CG, ^*^ *P* < 0.05.

To further validate the relationship between miR‐505 and HMGB1, the downstream target gene of miR‐505 was predicted by Targetscan7.2, and target binding loci were found between the 2. The dual‐luciferase activity of cells with HMGB1 3′ UTR‐Wt reduced significantly (*P* < 0.001) owing to miR‐505 overexpression which, however, had little influence of the same of HMGB1 3′ UTR‐Mut (*P* < 0.001). Moreover, WB showed a sharp decrease in the expression of HMGB1 protein in MG‐63 and U20S cells after transfection of miR‐505 mimics (*P* < 0.05; Fig. 5).

## Discussion

The treatment of osteosarcoma has developed into systemic chemotherapy and local control surgery. Although these methods have preliminarily increased the survival rate of patients with osteosarcoma, little progress has been made in this field in recent years [21, 22, 23]. Therefore, the exploration of the onset mechanism of osteosarcoma and the discovery of new targets for treatment play an important role in the improvement of prognosis of patients with osteosarcoma.

In this study, we observed the expression of miR‐505 and HMGB1 and analyzed their clinical values in osteosarcoma. Based on the expression of both groups, it was shown that miR‐505 expression was significantly lower in cancer tissues than that in paracancerous tissues and in the serum in the SG than that in the CG; a contrasting result was obtained for HMGB1 expression. Pearson’s correlation analysis revealed that the expression of miR‐505 and HMGB1 in tissue was positively associated with that in the serum, whereas the two indexes were negatively associated in tissue. ROC demonstrated that the AUC of miR‐505 in tissues was < 0.95 and that of HMGB1 was < 0.90. These results indicate that serum miR‐505 and HMGB1 have a better diagnostic efficacy in patients with osteosarcoma. Next, the relationship between miR‐505, HMGB1, and the patients' pathological data was further analyzed. It was demonstrated that miR‐505 and HMGB1 were closely associated with TNM staging, tumor size, and distant metastasis. Accordingly, it was suggested that miR‐505 and HMGB1 participate in the development and progression of osteosarcoma, and this was further validated through cytobiological tests.

A number of previous studies have focused on the biological functions of miR‐505 and HMGB1 in human diseases. Indeed, a study conducted by Ren *et al*. [24] found that miR‐505 was downregulated in liver cancer tissues and cell clones because the overexpression of miR‐505 suppressed the proliferation of liver cancer cells. Studies have shown that in necrotic cells, HMGB1 is released from the cells and induces a pro‐inflammatory response, which acts as DAMP in passive release of necrotic cells [25]. Molecular studies have demonstrated that miR‐505 can target IGF‐1R and inhibit its expression, resulting in the suppression of the AKT/GLUT1 channel and glycolysis of liver cancer cells. In the study conducted by Ding *et al*. [26], HMGB1 activated immunological cells to produce pro‐inflammatory factors, which accounted for damage to the epithelial barrier and organ functions and even death. In the current study, the detection of miR‐505 and HMGB1 in normal human bone cells and osteosarcoma cells found that miR‐505 expression was significantly lower in osteosarcoma cells than in normal human bone cells, whereas HMGB1 expression was significantly higher. Subsequently, the cell clones with more significant difference were transfected with miR‐505 mimics, miR‐NC, si‐HMGB1, and si‐NC to observe the biological functions of the cells. By transfecting MG‐63 and U20S cells with plasmids of miR‐505 mimics and si‐HMGB1, it was found that the high expression of miR‐505 can inhibit cell proliferation and invasion and promote apoptosis; this finding was in agreement with that of previous studies. Moreover, the suppression of HGMB1 expression resulted in obvious suppression of cell proliferation and invasion capacities and elevated the apoptosis rate, indicating that miR‐505 and HMGB1 can be used as potential targets of osteosarcoma. To explore the specific channels through which miR‐505 and HMGB1 affect the biological functions of osteosarcoma cells, we visited the online target gene prediction website (http://www.targetscan.org/vert_72/) and found that there were binding targets between HMGB1 and miR‐705. According to co‐transfection experiments, the consequences of miR‐505 overexpression and suppressed expression of HMGB1 significantly reduced the proliferation and invasion capacities of MG‐63 and U20S, and a decreased apoptosis rate indicated a possible close association between miR‐505 and HMGB1. Consequently, we used the dual‐luciferase reporter system; the results demonstrated that the dual‐luciferase activity of HMGB1 3' UTR‐Wt was significantly reduced owing to miR‐505 overexpression which, however, had little influence of the same of HMGB1 3′ UTR‐Mut. WB revealed that the expression of HMGB1 protein was significantly reduced after transfection with miR‐505 mimics, indicating the existence of a target regulation relationship between miR‐505 and HMGB1.

This study has a number of limitations. First, we did not perform experiments with nude mice, and this prevented the analysis of the results of miR‐505 injection to the tumor in mouse. Second, there was an insufficient focus on the signal channel to further analyze the relationships among miR‐505, HMGB1, and the signal channel as well as its impact on the biological functions of osteosarcoma cells, leading to incomplete results. These limitations should be incorporated into future studies to fully validate the results of the current study.

## Conclusions

In conclusion, miR‐505 overexpression has the potential to be used as a clinical index and target because it inhibits HMGB1 expression as well as the proliferation and invasion capacity of osteosarcoma cells, while also increasing the apoptosis rate.

## Conflict of interest

The authors declare no conflict of interest.

## Author contributions

GL and YH conceived the study and designed the experiments. FL and JM contributed to the data collection, performed the data analysis, and interpreted the results. GL wrote the manuscript; and YH revised the manuscript. All authors read and approved the final manuscript.
